# Cell-line dependency in cerebral organoid induction: cautionary observations in Alzheimer’s disease patient-derived induced pluripotent stem cells

**DOI:** 10.1186/s13041-022-00928-5

**Published:** 2022-05-16

**Authors:** Ju-Hyun Lee, Geon Yoo, Juhyun Choi, Si-Hyung Park, Hyogeun Shin, Renuka Prasad, Yeunehee Lee, Mee Ryung Ahn, Il-Joo Cho, Woong Sun

**Affiliations:** 1 Department of Anatomy, Brain Korea 21 Plus Program for Biomedical Science, Korea University College of Medicine, 73, Inchon-ro, Seongbuk-gu, Seoul, 02841 Republic of Korea; 2 Clinical Research Division, National Institute of Food and Drug Safety Evaluation, Ministry of Food and Drug Safety, Cheongju, Chungcheongbuk-do 28159 Republic of Korea; 3 Center for BioMicrosystems, Brain Science Institute, Korea Institute of Science and Technology (KIST), Seoul, 02792 Republic of Korea; 4 School of Electrical and Electronics Engineering, Yonsei University, Seoul, 03722, Republic of Korea

**Keywords:** Cerebral organoid, Disease modeling, Alzheimer’s diseases, Cell-to-cell variation

## Abstract

The cerebral organoid (CO) model has been used in the study of various neurodegenerative diseases owing to its physiological implications. However, the CO model may only be representative of certain clinical findings in affected patients, while some features are not recapitulated. In this study, we found that neurons in the CO model from patients with Alzheimer’s disease were less responsive to depolarization, in contrast to previous reports. This difference may be partly attributed to the variations in brain spatial identity depending on the genetic background of the induced pluripotent stem cells. Our current observation raises concerns that the phenotypes observed in the CO model need to be carefully evaluated for their clinical implications.

**Supplementary Information:**

The online version contains supplementary material available at 10.1186/s13041-022-00928-5.

The advent of cerebral organoid (CO) culture technology has received great attention as a better model for studying human brain diseases [1]. The CO model is based on spontaneous neural induction during the 3D culture of human pluripotent stem cells. The extended culture of COs allows the achievement of brain-like histoarchitectures, diverse cell compositions, and embryonic/neonatal brain-like neural activities [1–4]. These 3D CO models can successfully reproduce many aspects of human-specific brain development and related developmental brain pathologies. Furthermore, many attempts have been made to model late-onset neurodegenerative diseases, such as Alzheimer’s disease (AD) [5–8]. For instance, COs produced from AD patient-derived human induced pluripotent stem cells (iPSCs) exhibited enhanced accumulation of amyloid β (Aβ), neurodegeneration, and hyperactivation of neurons [5–8]. Some features correlate well with histopathology, such as Aβ deposits. However, some features, such as neuronal hyper-activation, are relatively unclear. Thus, the significance of the observations made from CO models should be carefully examined.

To utilize COs for modeling AD, we obtained normal and familial AD patient-derived iPSCs that have been validated with known AD-related phenotypes [9]. Both types of iPSCs were readily stained by alkaline phosphatase (AP), and major stemness factors such as NANOG, OCT4, and SOX2 were similarly expressed in all cells in the colonies; this finding suggested that the cells maintained their stemness equally (Additional file 1: Fig. S1). However, when COs were generated using Lancaster’s protocol [1, 10], the organoids from AD iPSCs were significantly larger than those from normal iPSCs (Fig. 1A and B). Furthermore, a considerably larger proportion of AD-derived organoids exhibited dark spots, which are known to originate from spontaneous induction of the retinal field (Fig. 1C) [1]. Owing to a high degree of histological variability in the individual organoids, the cell line-related differences in the organoids were difficult to evaluate (Fig. 1D). However, the time course of the reduction in expression of the stemness marker (OCT4), expression of the neural stem cell marker (SOX2), and induction of the neuronal marker (TUJ1) was not significantly different between the two groups, indicating similar neuronal maturation (Fig. 1E). In contrast, the expression level of regional identity markers showed that AD iPSC-derived organoids exhibited more forebrain markers (FOXG1 and PAX6) and fewer midbrain markers (LMX1A and EN1) (Fig. 1F and Additional file 1: Fig. S2). Moreover, AD organoids exhibited stronger expression of the retinal marker RAX; this gene expression pattern is consistent with the abundance of melanin spots detected in AD iPSC-derived organoids (Fig. 1A and C). Collectively, it appeared that two different organoids exhibit different mixtures of regional identity.

**Fig. 1:**
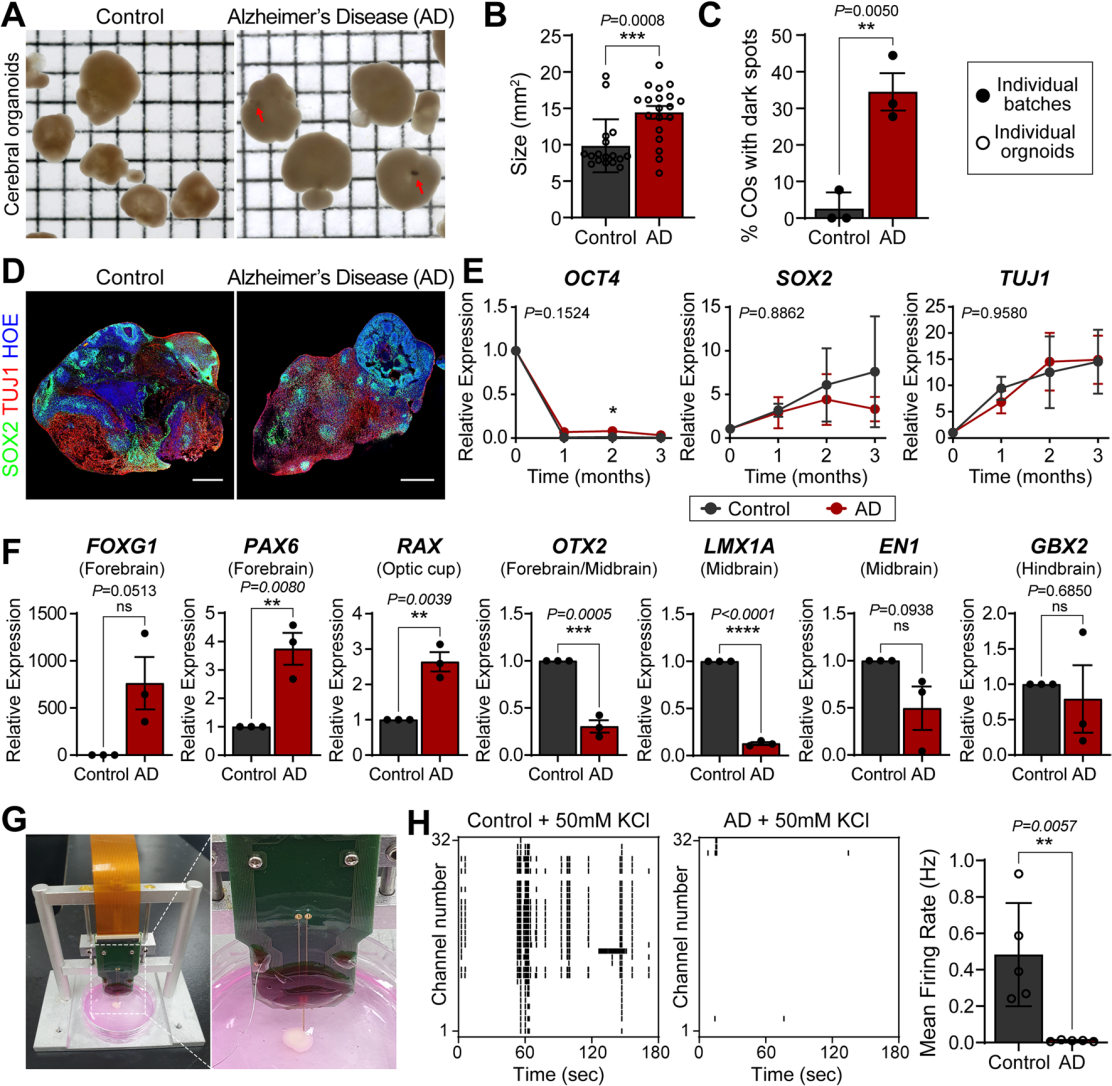
Characterization of COs from normal and familiar AD patient-derived iPSCs. **A** Bright-field images of 3-month-old COs. Red arrows indicate the dark spots in COs. Square units, x: 2 mm, y: 2 mm. **B** Quantification of individual CO size. Data are presented as mean ± SEM (two independent experiments; n = 17 for control; n = 19 for AD). **C** Proportion of COs exhibiting dark spots. Data were obtained from three independent experiments, and presented as mean ± SEM. **D** Immunostaining for SOX2 (neural progenitors, green) and TUJ1 (neurons, red). Nuclei were counterstained with Hoechst (blue). Scale bar, 500 μm. **E** Real-time PCR profiles of gene expression for pluripotency (OCT4), neural induction (SOX2), and neuronal differentiation (TUJ1). Nuclei were counterstained with Hoechst (blue). Data were obtained from three independent experiments, and presented as mean ± SEM. *P < 0.05. **F** Comparison of regional identity of 2-month-old COs. Relative expression of the region-specific neural progenitor markers: forebrain (FOXG1, PAX6, and OTX2), optic cup (RAX), midbrain (OTX2, LMX1A, and EN1), and hindbrain (GBX2). Data were obtained from three independent experiments, and, presented as mean ± SEM. **G** MEA system with a microdrive for the electrophysiological recording of the COs. **H** The representative plot from KCl-mediated neural activities recorded in 3-month-old COs. Bar graphs show the mean firing rate of individual CO. Data were obtained from an independent-measures experiment (n = 5 for control and n = 5 for AD), and presented as mean ± SD. P-value is determined using unpaired t-test (**B**, **C**, **F**, and **H**) or two-way ANOVA followed by Bonferroni’s multiple comparisons test (**E**)

To address whether these differences are associated with AD-related pathological symptoms, we examined several phenotypes associated with AD symptoms. First, the expression levels of AD-related genes (MAPT and APP) were similar between the two groups (Additional file 1: Fig. S3). This finding suggests that the 3-month culture may be insufficient to demonstrate the histopathological hallmarks of AD. On the contrary, microneural probe-based electrophysiological recordings demonstrated that KCl-induced neuronal excitation was greatly reduced in the AD group compared to the control group (Fig. 1G and H). This result is in sharp contrast to the previous observation that neurons derived from AD iPSCs are hyperexcitable [7, 8]. These results indicated that the detected differences between the two groups did not correlate with AD-related phenotypes.

These discrepancies are theoretically derived from several causes, including collective genetic variations (genetic backgrounds), disease-causing mutations, and non-genetic factors such as batch variations. Of these potential causes, batch variations were excluded because the results from three independent trials were consistent. Considering that the previously reported phenotypes were not reproduced in our experiments [7, 8], we also ruled out the possibility that these discrepancies were primarily caused by the disease-causing mutation in AD patient-derived iPSCs. Intrinsic differences in the developmental signaling pathways among iPSCs have been appreciated [11], and thus we speculated that innate differences (genetic variations) in two iPSCs may strongly affect the development of COs. To circumvent this potential risk, recent studies for disease modeling have used isogenic controls produced by gene editing [5, 7, 8]. However, it is noteworthy that the genetic background can greatly affect the development and pathophysiological phenotypes that are often considered AD-related symptoms. In addition, spontaneous and undirected induction of COs results in relatively large variations [3, 12]. Therefore, the guided neural organoid induction protocols often use different concentrations of the regional inducing factors in each cell line to obtain the same regional identity [13–15]. Thus, with current protocols that utilize the spontaneous induction mechanism, organoids from different iPSCs appear to show different distribution of the region-specific neural parts, which primarily contributed to the regionalization and neural circuit development during the growth of COs.

Although it is true that the current observation is only anecdotal, and larger scale examination is required, genetic variations appear to affect organoid development and maturation more substantially than we assume. Thus, caution is needed to verify the value of the readouts. In particular, whether these differences are selectively linked to the AD-specific gene mutations, and whether the observed differences in organoid models are indeed causally associated with AD-specific pathologies must be considered.

## Materials and methods

### iPSC culture

Human iPSCs (UCSD065i-20-3 and UCSD239i-APP2-1) were purchased from the WiCell Research Institute. The human iPSCs were maintained on Matrigel (BD Biosciences, 354277)-coated plates in mTeSR1 (STEMCELL Technologies, 85850). The human iPSCs were maintained under 5% CO_2_ at 37 °C with daily medium changes. The human iPSCs were passaged every 5 days into small clumps using ReLeSR (STEMCELL Technologies, 05872) and replated onto pre-coated culture dishes. All experiments were performed on human iPSCs with less than 40 passages.

### Generation of cerebral organoids

The generation of human COs was performed according to the previously described protocol [10]. Briefly, hiPSC colonies were dissociated using Accutase (Innovative Cell Technologies, AT-104). To generate embryoid bodies (EBs), dissociated cells were seeded onto a 96-well low-attachment plate (9000 cells per well) in low-bFGF hESC medium with the ROCK inhibitor. The EBs were cultured for an additional 5–6 days until they grew to 400 μm in diameter. The culture conditions were changed to induce primitive neuroepithelia for 4–5 days. When the EBs exhibited a radial arrangement of neuroepithelia, they were embedded in Matrigel droplets and transferred to a neural differentiation medium without vitamin A. After 4 days, the Matrigel-embedded EBs were transferred to an orbital shaker and grown in a neural differentiation medium containing vitamin A.

### AP staining

AP staining was performed using an alkaline phosphatase detection kit (Merck, SCR004), according to the manufacturer’s instructions. Briefly, the cultured iPSCs were fixed in 4% paraformaldehyde (PFA; Biosesang) for 2 min at room temperature and washed two times in TBST (0.05% Tween-20). The samples were incubated in AP staining solution for 15 min in the dark at room temperature and washed two times with TBST. Images were acquired with an EVOS 5000 microscope (Life Technologies).

### Immunofluorescence

The COs were fixed by immersion in 4% PFA overnight at 4 °C and washed several times with PBS. The samples were then incubated in 30% sucrose in PBS at 4 °C until completely submersed, embedded in Tissue-Tek Optimal Cutting Temperature (O.C.T.) compound (SAKURA), frozen on dry ice, cryo-sectioned serially at 16–40 μm thickness, and collected onto New Silane III coating slides (Muto Pure Chemicals Co. Ltd, 5118-20F). For immunostaining, the samples on slides were washed with PBS three times for 5 min each at room temperature, blocked with a solution (3% BSA, 0.2% Triton X-100 in PBS) for 30 min at room temperature, and then incubated with the respective primary antibody diluted in blocking solution overnight at 4 °C. The antibodies used in this study were rabbit anti-SOX2 (1:500, Millipore, AB5603) and mouse anti-beta-tubulin III (TUJ1; 1:1000, Sigma, T8660). Subsequently, samples were washed with PBS three times for 5 min each at room temperature and then incubated with the respective secondary antibody and Hoechst33342 diluted in blocking solution for 30 min at room temperature. The secondary antibody was subsequently washed with PBS, and the samples were mounted on Crystal Mount (Biomeda, M02). All steps were performed with gentle shaking. Images were captured and processed using a Leica TCS SP8 confocal microscope.

### Real-time PCR analysis

Total RNA was isolated from organoids using TRIzol™ Reagent (Invitrogen, 15596-026) in triplicate according to the manufacturer’s instructions. The isolated RNA (1 μg) was used to synthesize cDNA using murine Moloney leukemia virus reverse transcriptase (Promega, M5313). cDNA was then amplified using gene-specific primers (primer sequences are listed in Additional file 1: Table S1). Real-time PCR (Applied Biosystems, ABI7500) analysis was performed using the SYBR Green master mix (Enzynomics, RT500S) in combination with specific primers. Reactions were performed using an Eppendorf Realplex4 cycler (Eppendorf). All values were normalized to GAPDH expression for calculating the fold change.

### MEA analysis

To evaluate the functionality of cultured 3-month-old COs, we used a MEMS neural probe integrated with 32 black Pt microelectrodes for neural signal recording [16]. The COs were transferred from the culture dish to a 35-mm Petri dish-based recording chamber. After positioning the COs under the neural probe, the sample was embedded in low-melt agarose, and the neural probe was slowly inserted into the COs via the microdrive. The recording chamber was filled with a fresh culture medium. To measure the neural activity by depolarization, we directly treated 50 mM KCl in the recording chamber. Neuronal activity was recorded for at least 5 min in each sample. The recorded signals were processed and digitized using an RHD2132 amplifier board connected to an RHD2000 Evaluation System (20 kS/s per channel, 300 Hz high-pass filter, 6 kHz low-pass filter, and 16-bit ADC). To analyze the recorded neural activity, the recorded neural signals were sorted using a custom MATLAB sorting algorithm [16].

### Statistical analysis

Statistical analyses were performed using an unpaired Student’s t-test to compare two groups, and with a two-way ANOVA with Bonferroni’s multiple comparisons test for multiple comparisons. All analyses were performed using GraphPad Prism 9 software. The results are presented as the mean ± SEM or SD. Statistical significance was set at P < 0.05.

## Supplementary Information

**Additional file 1: Figure S1.** Pluripotency of normal and familiar AD patient-derived iPSCs. **Figure S2.** Variable outcomes in neural induction of normal and AD patient-derived COs. **Figure S3.** Verification of AD-related gene expression. **Table S1.** Primer sequences used for real-time PCR.

## Data Availability

Datasets are available from the corresponding author on reasonable request.

## Abbreviations

3D: 3-Dimensional
AD: Alzheimer’s disease
AP: Alkaline phosphatase
A$$\beta$$: Amyloid beta
bFGF: Basic fibroblast growth factor
BSA: Bovine Serum Albumin
CO: Cerebral organoid
EB: Embryoid body
iPSC: Induced pluripotent stem cell
KCl: Potassium chloride
MEA: Multi-electrode array
MEMS: Micro-electromechanical systems
OCT: Optimal Cutting Temperature
PBS: Phosphate-buffered saline
PCR: Polymerase chain reaction
PFA: Paraformaldehyde
Pt: Platinum
