# Supplementary material for: Cell-line dependency in cerebral organoid induction: cautionary observations in Alzheimer’s disease patient-derived induced pluripotent stem cells
Source: Mol Brain. 2022 May 16;15:46. doi: 10.1186/s13041-022-00928-5 (PMC9109296; doi:10.1186/s13041-022-00928-5)
Supplement: Supplementary file 1 — Additional file 1: Figure S1. Pluripotency of normal and familiar AD patient-derived iPSCs. Figure S2. Variable outcomes in neural induction of normal and AD patient-derived COs. Figure S3. Verification of AD-related gene expression. Table S1. Primer sequences used for real-time PCR. [file 13041_2022_928_MOESM1_ESM.pdf]

Supplementary Information

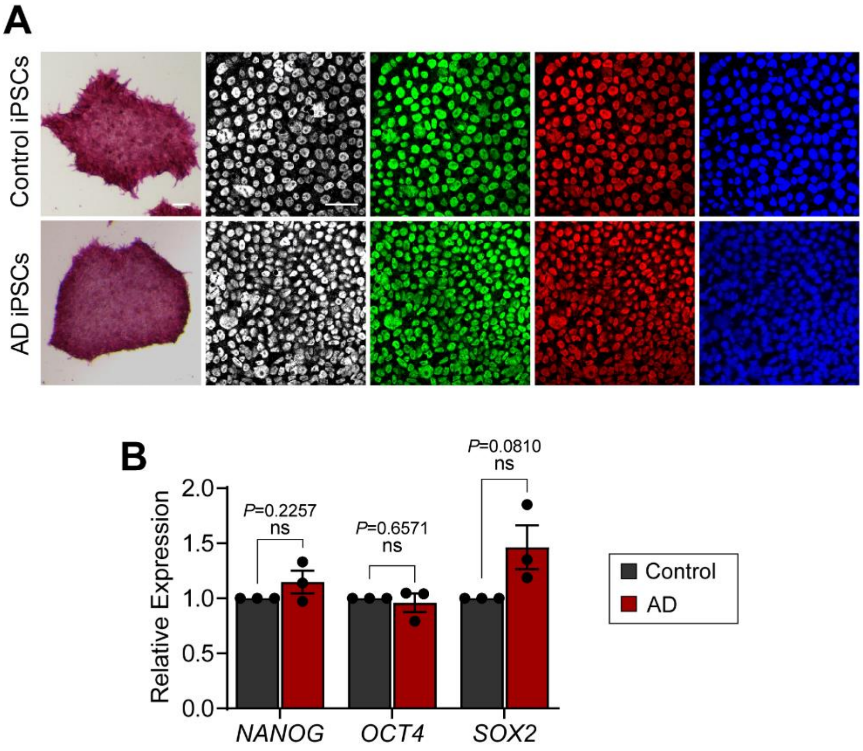

Figure S1. Pluripotency of normal and familiar AD patient-derived iPSCs

A. Alkaline phosphatase (AP) staining and immunocytochemistry of the two types of iPSCs. Scale bar, 50 μm. **B.** Relative expression of pluripotent genes (NANOG, OCT, and SOX2). Data were obtained from three independent experiments, and presented as mean ± SEM. P-value is determined using unpaired t-test.

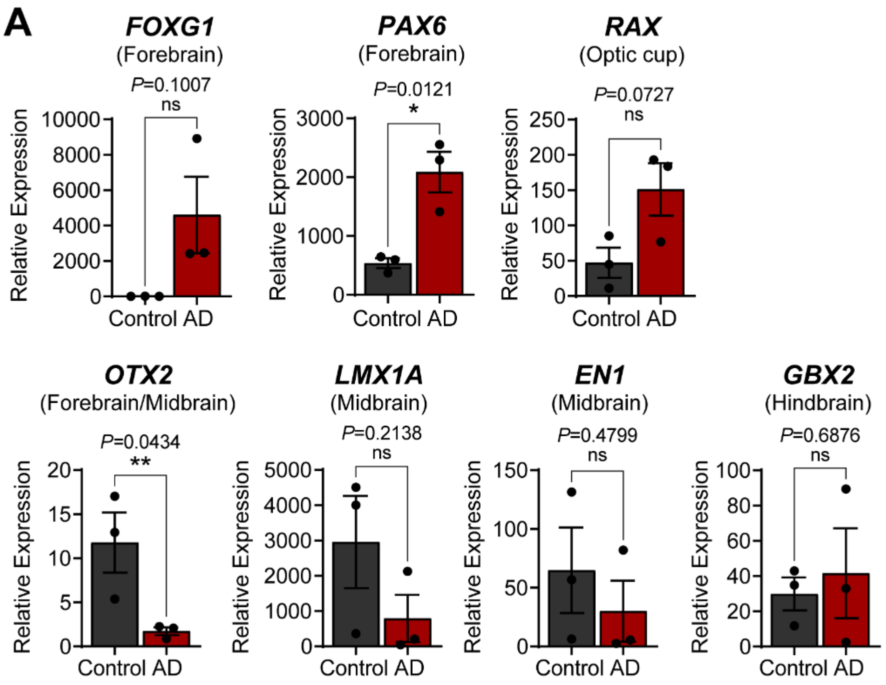

Figure S2. Variable outcomes in neural induction of normal and AD patient-derived COs.

A. Comparison of regional identity of 2-month-old COs. Relative expression of the region-specific neural progenitor markers: forebrain (FOXG1, PAX6, and OTX2), optic cup (RAX), midbrain (OTX2, LMX1A, and EN1), and hindbrain (GBX2). The expression levels of all analyzed genes were normalized to the same gene expression for each iPSC sample. Data were obtained from three independent experiments, and presented as mean ± SEM. P-value is determined using unpaired t-test.

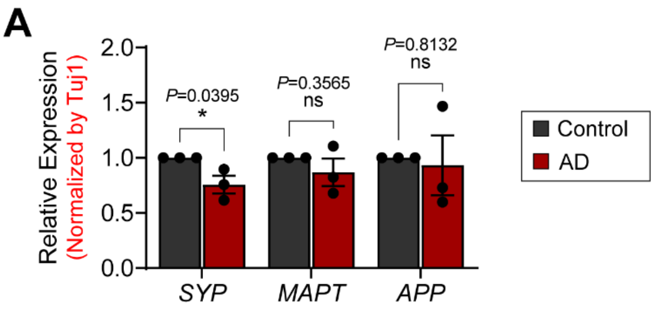

Figure S3. Verification of AD-related gene expression.

A. Relative expression of synapse formation (SYP) and AD-related genes (MAPT and APP). Expression levels of each gene were normalized to the TUJ1 expression level. Data were obtained from three independent experiments, and presented as mean ± SEM. P-value is determined using unpaired t-test.

Supplementary Table 1. Primer sequences used for real-time PCR

Gene	Primer (Forward)	Primer (Reverse)	Size
OCT4	CTGGTTCGCTTTCTCTTTCG	CTTTGAGGCTCTGCAGCTTA	150
NANOG	AAGGCCTCAGCACCTACCTA	TGCACCAGGTCTGAGTGTTC	181
SOX2	GGAAAGTTGGGATCGAACAA	GCGAACCATCTCTGTGGTCT	145
OTX2	CCAGACATCTTCATGCGAGAG	GGCAGGTCTCACTTTGTTTTG	147
TUJ1 (TUBB3)	GCTCAGGGGCCTTTGGACATCTCTT	TTTTCACACTCCTTCCGCACCACATC	148
FOXG1	CCGCACCCGTCAATGACTT	CCGTCGTAAAACTTGGCAAAG	133
PAX6	TCCGTTGGAACTGATGGAGT	TAAGGATGTTGAACGGGCAG	146
RAX	AAGCCCCTCGACCCTACTG	CCGCCGATGCTTTTTCTTGG	128
OTX2	CCAGACATCTTCATGCGAGAG	GGCAGGTCTCACTTTGTTTTG	147
LMX1A	GCAAAGGGGACTATGAGAAGGA	CGTTTGGGGCGCTTATGGT	160
EN1	GAGCGCAGGGCACCAAATA	CGAGTCAGTTTTGACCACGG	92
GBX2	CTCACCTCTACGCTCATGGC	GCCTTGTCGAAGTTACCGC	125
SYP	CTCGGCTTTGTGAAGGTGCT	CTGAGGTCACTCTCGGTCTTG	122
MAPT	CCAAGTGTGGCTCATTAGGCA	CCAATCTTCGACTGGACTCTGT	106
APP	TCTCGTTCCTGACAAGTGCAA	GCAAGTTGGTACTCTTCTCACTG	116
GAPDH	CATCACTGCCACCCAGAAGACTG	ATGCCAGTGAGCTTCCCGTTCAG	153
